# T Cell Activators Exhibit Distinct Downstream Effects on Chimeric Antigen Receptor T Cell Phenotype and Function

**DOI:** 10.4049/immunohorizons.2400008

**Published:** 2024-06-10

**Authors:** Sarah Underwood, Jianjian Jin, Lipei Shao, Michaela Prochazkova, Rongye Shi, Hannah W. Song, Ping Jin, Nirali N. Shah, Robert P. Somerville, David F. Stroncek, Steven L. Highfill

**Affiliations:** * Center for Cellular Engineering, Department of Transfusion Medicine, Clinical Center, National Institutes of Health, Bethesda, MD; † Pediatric Oncology Branch, National Cancer Institute, National Institutes of Health, Bethesda, MD

## Abstract

T cell activation is an essential step in chimeric Ag receptor (CAR) T (CAR T) cell manufacturing and is accomplished by the addition of activator reagents that trigger the TCR and provide costimulation. We explore several T cell activation reagents and examine their effects on key attributes of CAR T cell cultures, such as activation/exhaustion markers, cell expansion, gene expression, and transduction efficiency. Four distinct activators were examined, all using anti-CD3 and anti-CD28, but incorporating different mechanisms of delivery: Dynabeads (magnetic microspheres), TransAct (polymeric nanomatrix), Cloudz (alginate hydrogel), and Microbubbles (lipid membrane containing perfluorocarbon gas). Clinical-grade lentiviral vector was used to transduce cells with a bivalent CD19/CD22 CAR, and cell counts and flow cytometry were used to monitor the cells throughout the culture. We observed differences in CD4/CD8 ratio when stimulating with the Cloudz activator, where there was a significant skewing toward CD8 T cells. The naive T cell subset expressing CD62L^+^CCR7^+^CD45RA^+^ was the highest in all donors when stimulating with Dynabeads, whereas effector/effector memory cells were highest when using the Cloudz. Functional assays demonstrated differences in killing of target cells and proinflammatory cytokine secretion, with the highest killing from the Cloudz-stimulated cells among all donors. This study demonstrates that the means by which these stimulatory Abs are presented to T cells contribute to the activation, resulting in differing effects on CAR T cell function. These studies highlight important differences in the final product that should be considered when manufacturing CAR T cells for patients in the clinic.

## Introduction

T cell activation is an essential step in chimeric Ag receptor (CAR) T cell manufacturing and research (1). Primary T cell activation occurs via three distinct signals. The first and second signals occur through occupancy of the TCR/CD3 complex and costimulation through CD28, whereas the third signal may be provided by cytokines (e.g., IL-2), which direct and amplify T cell differentiation and expansion (2–4). When using a mixed cell population of starting material, such as PBMCs, the costimulatory signal (CD28) may be delivered by APCs within this mixture. However, most cell therapy manufacturing centers are now electing to use selected T cells as their starting material to increase consistency of the culture and standardize the manufacturing process. Under these circumstances, APCs are not present, and the CD28 signal must be delivered separately. To deliver the appropriate signals for T cell activation, several reagents have been developed that provide signals 1 and 2 using anti-CD3 and anti-CD28 on a bead-based platform or scaffold, whereas signal 3 (usually IL-2, IL-7, or IL-15) is provided exogenously within the culture medium.

In this report, we examine the expansion characteristics, cell phenotype, transduction efficiency, and functional aspects of CD19/CD22 bivalent CAR T cells after manufacturing using four distinct T cell activation reagents. These include a magnetic microsphere platform (CTS Dynabeads; Thermo Fisher), a polymeric nanometrix platform (MACS GMP T Cell TransAct; Miltenyi Biotec), a perfluorocarbon gas-filled micelle platform (Microbubbles; Diagnologix), and an alginate hydrogel platform (Cloudz; R&D Systems). Dynabeads consist of superparamagnetic spherical, inert, polystyrene beads of uniform size of ∼4.5 μM in diameter conjugated with anti-CD3 and anti-CD28 Abs (5, 6). Dynabeads are removed from the culture by using a magnet. TransAct reagent is a colloidal polymeric nanomatrix conjugated to humanized anti-CD3 and anti-CD28 Abs (7). For TransAct, magnetic debeading is not required; the matrix is washed out during the culture process. Lipid Microbubble-conjugated anti-CD3 and anti-CD28 developed by Diagnologix have been shown to improve long-term expansion and reduce in vitro cell death of T cells compared with Dynabeads activation (8, 9). This reagent dissolves during the course of culture or can be removed earlier by applying hydrostatic pressure. Finally, the Cloudz Human T-Cell Activation kit developed by R&D Systems consists of dissolvable alginate-based hydrogel microspheres bound to anti-CD3 and anti-CD28 Abs. Table I summarizes key attributes of these reagents.

**Table I.** Summary of T cell stimulator details

Activator Name	Company	Catalog No.	Scalability	Good Manufacturing Practices Status	Short Description
CTS (Cell Therapy Systems) Dynabeads CD3/CD28	Life Technologies	40203D	1E9 cells/vial	Yes	Anti-CD3/ anti-CD28–coated magnetic beads
MACS GMP T Cell TransAct	Miltenyi Biotec	200-076-202	2E8 enriched T cells or up to 4E8 PBMCs/vial	Yes	Polymeric nanomatrix conjugated to anti-CD3/anti-CD28
GMP Cloudz Human T Cell Activation Kits (discontinued)	R&D Systems	CLD001-GMP	4E8 CD3^+^ T cells per kit	Yes	Microspheres composed of alginate-based hydrogel bound to anti-CD3/anti-CD28
Microbubbles	Diagnologix	Not Applicable	Not commercially available; 2E8 bubbles/ml	No	Perfluorocarbon gas with phospholipid membrane bound to anti-CD3/anti-CD28

In this study, we aimed to investigate differences in the final CD19/CD22 bivalent CAR T cell product because they may relate to the method by which these T cells were activated. The culture conditions were otherwise identical to minimize variability caused by other factors. We discovered differences in CAR T cell CD4/CD8 T cell ratios, T cell subset differentiation capacity, gene expression, and cytotoxicity. Cell therapy manufacturing centers and research laboratories should consider these differences when choosing one of these reagents for the production of CAR T cells.

## Materials and Methods

### T cell culture

PBMCs were collected by apheresis from healthy donors. CD4^+^ and CD8^+^ cells were selected from the PBMCs using good manufacturing practices–compliant Miltenyi CliniMACS Plus device and were cryopreserved. CD4^+^/CD8^+^ cells were thawed prior to use in thaw media containing AIM-V media (Life Technologies, Grand Island, NY) with 10% heat-inactivated human AB serum (Valley Biomedical, Winchester, VA), 10 U/ml heparin (Fresenius Kabi), and 10 μg/ml Dornase Alfa/Pulmozyme (Genentech). Cultures were initiated in bags with 40 × 10^6^ viable CD4^+^ and CD8^+^ cells in AIM-V media containing 5% heat-inactivated human AB serum, 2 mM Glutamax (Life Technologies), and 40 IU/ml IL-2 (Prometheus Laboratory, San Diego, CA). T cells in each group were activated with either Cloudz, Dynabeads, Microbubbles, or TransAct following the corresponding manufacturers’ standard procedures. All activators were left in culture until day 4, when they were removed, with the exception of Microbubbles, which spontaneously dissolve at ∼24 h in culture according to the manufacturer. GMP Cloudz Human T-Cell Activation Kit was obtained from R&D Systems. Dynabeads CD3/CD28 were obtained from Cell Therapy Systems. Microbubbles were obtained from Diagnologix. MACS GMP T Cell TransAct was obtained from Miltenyi Biotec. Unactivated control T cells were placed in the same media without the use of stimulators.

Cultures were transduced with clinical-grade bivalent CD19/CD22 lentiviral vector on day 2 at 40 or 80 multiplicity of infection (MOI). On day 4, transduction was stopped, and activation reagents were removed if necessary (Dynabeads and Cloudz). Culture media were changed to the same media except with 100 IU/ml IL-2, and cells were diluted to 0.5 × 10^6^ viable WBCs/ml.

Cell count and viability were measured on the Cellometer instrument (Nexcelom Biosciences) using Acridine Orange/Propidium Iodide staining. Cell counts and flow cytometry were performed on days 0, 2, 4, and 7. Cells were harvested on day 7, and final aliquots were cryopreserved using CryoStor CS10 (BioLife Solutions) for future functional assays. Samples for RNA analysis were lysed and frozen for future gene expression studies.

### Lentiviral vector

The lentiviral vector was designed to incorporate a CAR consisting of two single-chain variable fragments (scFvs) targeting CD19 and CD22, a hinge region, a transmembrane region, and intracellular regions of human 41BB and CD3-ζ molecules. These two scFvs, FMC63 for CD19 CAR and m971 for CD22 CAR, have also been in use in phase I/II clinical trials as separate entities at the National Institutes of Health (NIH) Clinical Center and elsewhere. The current bivalent CAR combines these scFvs into one CAR construct, where the CD22 scFv was placed in a membrane-distal location and the CD19 CAR was placed in a membrane-proximal position. This CAR also incorporated a short G4Sx1 linker between the CD19 and CD22 scFvs, which was found to improve detection and function (10). This CAR is encoded by a self-inactivating lentiviral vector and is designated MSCV-CAR1922-WPRE. The vector was prepared and preserved following current good manufacturing practices conditions at Lentigen Technology (a Miltenyi Biotec Company).

### Flow cytometry

Flow cytometry was used to evaluate phenotype, activation and exhaustion, T cell subsets, transduction efficiency, and cytotoxicity. Cells were resuspended in PBS (Life Technologies) plus 0.5% human serum albumin (Baxalta), stained for 30 min at 4°C, then washed once. Samples were analyzed on the Cytoflex flow cytometer (Beckman Coulter). All Abs used and their sources are listed in Supplemental Table I. Dead cells were excluded from analysis by 7AAD (BD Biosciences) or Zombie Aqua (Invitrogen). Tumor cell percentage (in functional assays) was evaluated by flow cytometry in the FITC channel because NALM-6 cells were tagged with GFP. Flow gating is shown in Supplemental Figs. 1 and 2.

### Functional assays

Effector cell function was tested by coculturing with the target NALM-6 tumor cell line (ATCC) at ratios of 5:1 and 1:1 (E:T). Effector cells are defined in this article as CAR T cells that express CD19/CD22 bivalent CAR. Cryopreserved aliquots were thawed and cultured overnight before coculture was initiated. For each type of activator, untransduced T cell control samples and tumor cell control samples were prepared in triplicate and cultured for 24 and 48 h. Cytotoxicity was evaluated based on flow cytometry and cell counts using the Cellometer to calculate tumor cell and CAR T cell number per well. Culture medium was RPMI 1640 (Life Technologies) with 5% FBS (HyClone) for all cultures. Supernatants were frozen for future multiplex cytokine immunoassays on the Bio-Plex 200.

### Cytokine analysis

Frozen cell culture supernatants were transported to the laboratory and processed within 2 h. Frozen samples were thawed on ice and spun for 8 min at 10,000 × *g* in 4°C centrifuge to completely remove precipitates. Undiluted samples were assayed on a suspension multiplex array system (Bio-Plex 200 Systems; Bio-Rad, Hercules, CA) with a Bio-Plex Pro Human Cytokine Screening Panel, 48-Plex kit (catalog no. 12007283; Bio-Rad) following the manufacturer’s instructions (Bio-Rad). Samples were run in duplicate. Results were analyzed using the Bio-Plex Manager Software (version 6.2.0.175).

### Gene expression analysis

Gene expression was measured using the nCounter Analysis Pipeline (NanoString Technologies) with the CAR T cell characterization gene panel (n = 780 genes), and data were analyzed using nSolver and RStudio environment. ggplot2 (version 3.3.4) and pheatmap packages (version 1.0.10) were used for visualization. Pathway score was derived by calculating the first principal component of pathway genes’ normalized expression in the nSolver software with default parameters according to the manual (MAN-10030-03; NanoString). The algorithm was established by Tomfohr et al. (11) in 2005.

### Statistical analysis

**FIGURE 1.**
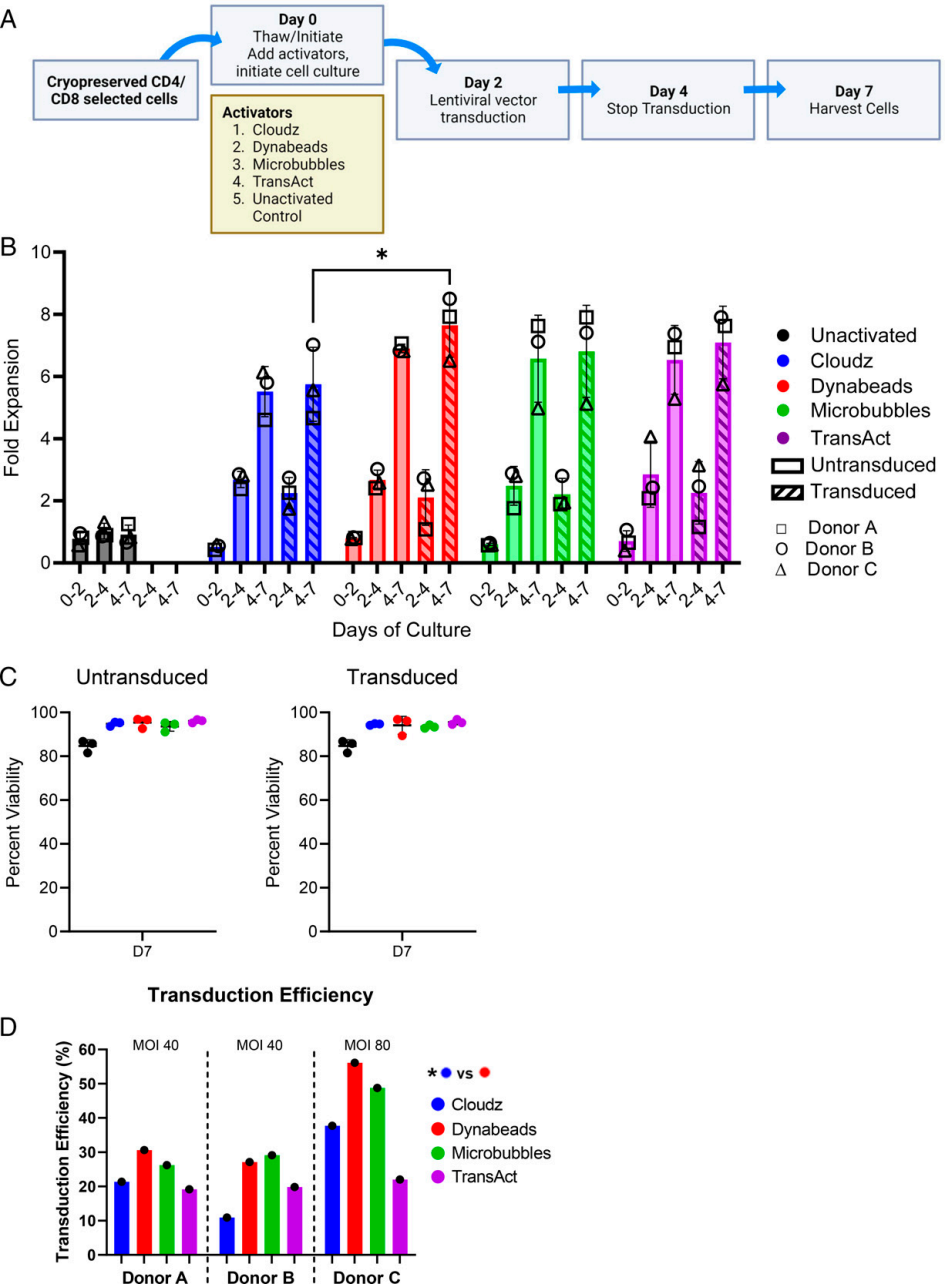
Fold expansion, viability, and transduction efficiency. (**A**) Schematic of 7-d culture used in this study. (**B**) Fold expansion for each day of culture was calculated by dividing the initial total nucleated cells (TNC) on the subsequent day by the final TNCs on the previous day. Untransduced and transduced groups are shown for all donors. (**C**) Viability is shown for day 7 for all groups and donors. (**D**) A vector MOI of 80 was used for donor C compared with 40 for donors A and B. For all sections, *p ≤ 0.05.

**FIGURE 2.**
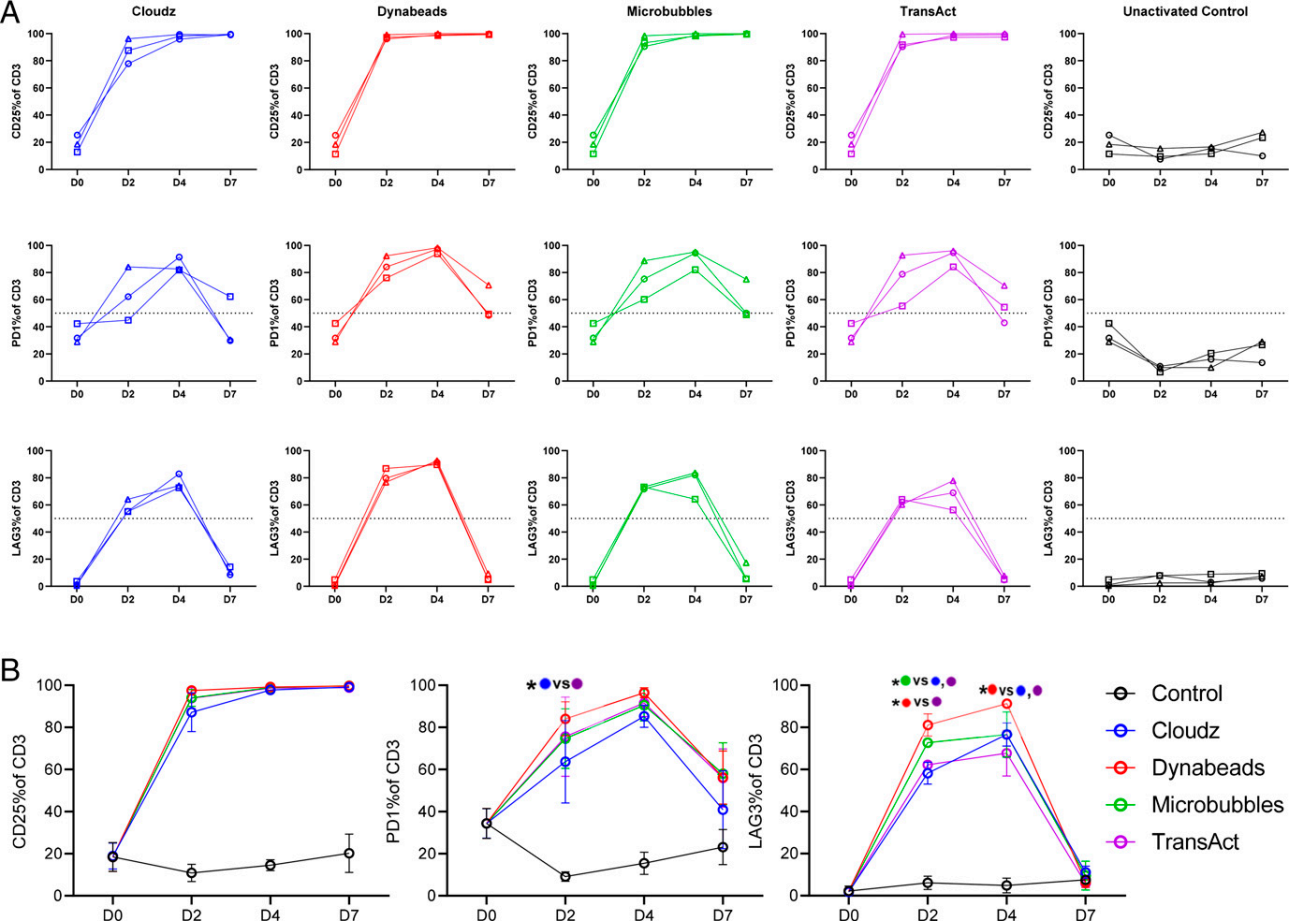
T cell activation and exhaustion markers. (**A**) Percent CD25^+^, PD1^+^, LAG3^+^ of CD3^+^ cells by flow cytometry is shown for the duration of culture for all transduced groups and the unactivated control group. Dotted line is shown at 50% for ease of viewing differences in PD1 and LAG3 expression. (**B**) Averages from (A) are shown overlaid for each day. Significant differences are shown for PD1^+^% on day 2 and LAG3^+^% on days 2 and 4. For all sections, *p ≤ 0.05.

**FIGURE 3.**
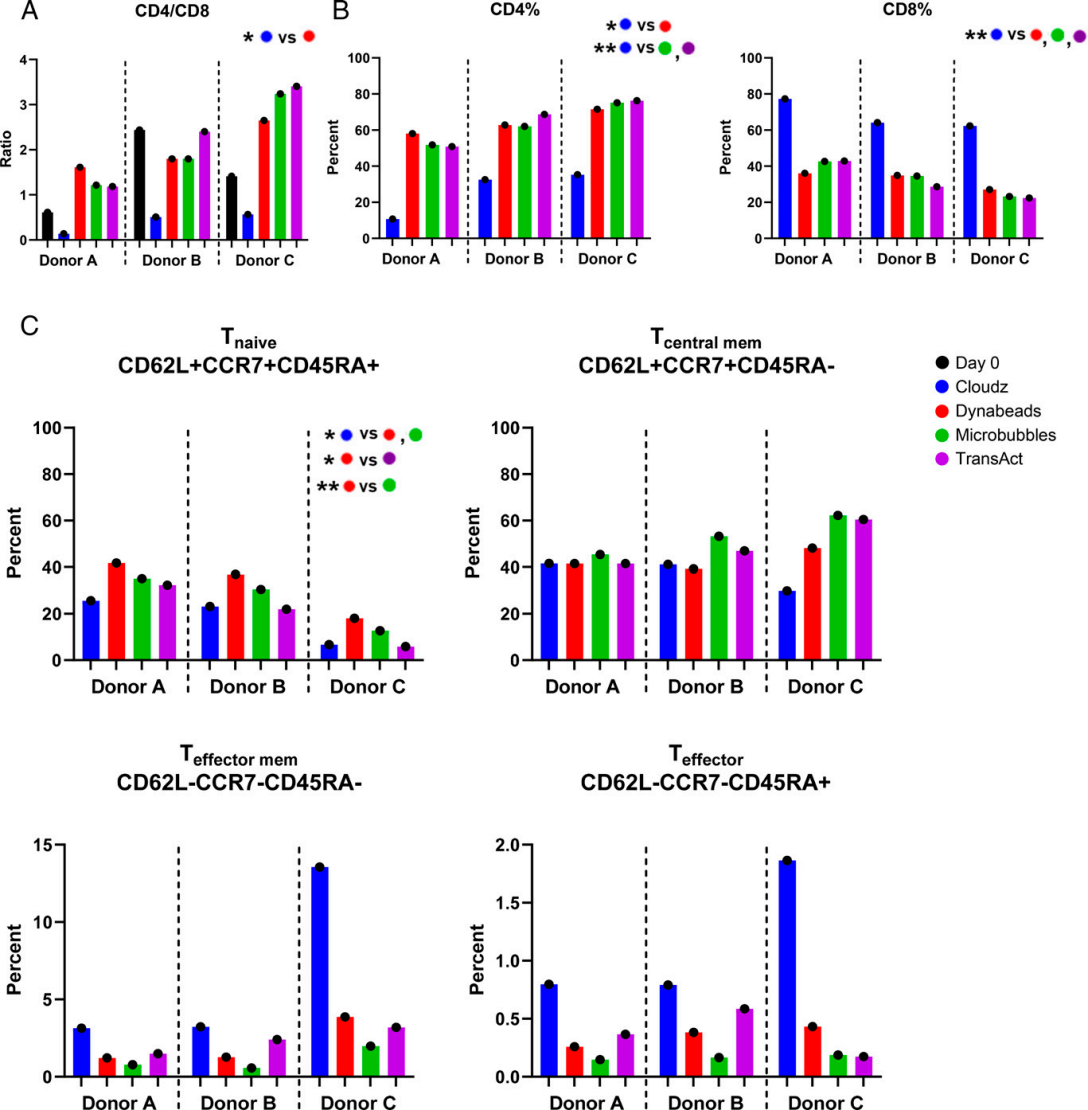
Effects of activators on T cell subsets. (**A**) CD4/CD8 ratio was calculated for all donors and groups on days 0 and 7 by dividing percent CD4^+^ by CD8^+^ of CD3^+^. (**B**) Day 7 frequencies of CD4^+^ (left) and CD8^+^ (right) of CD3^+^ cells are shown. (**C**) Frequencies of cells expressing markers associated with naive (upper left), central memory (upper right), effector memory (lower left), or T effector (lower right) cell populations on day 7. For all sections, *p ≤ 0.05, **p ≤ 0.01.

**FIGURE 4.**
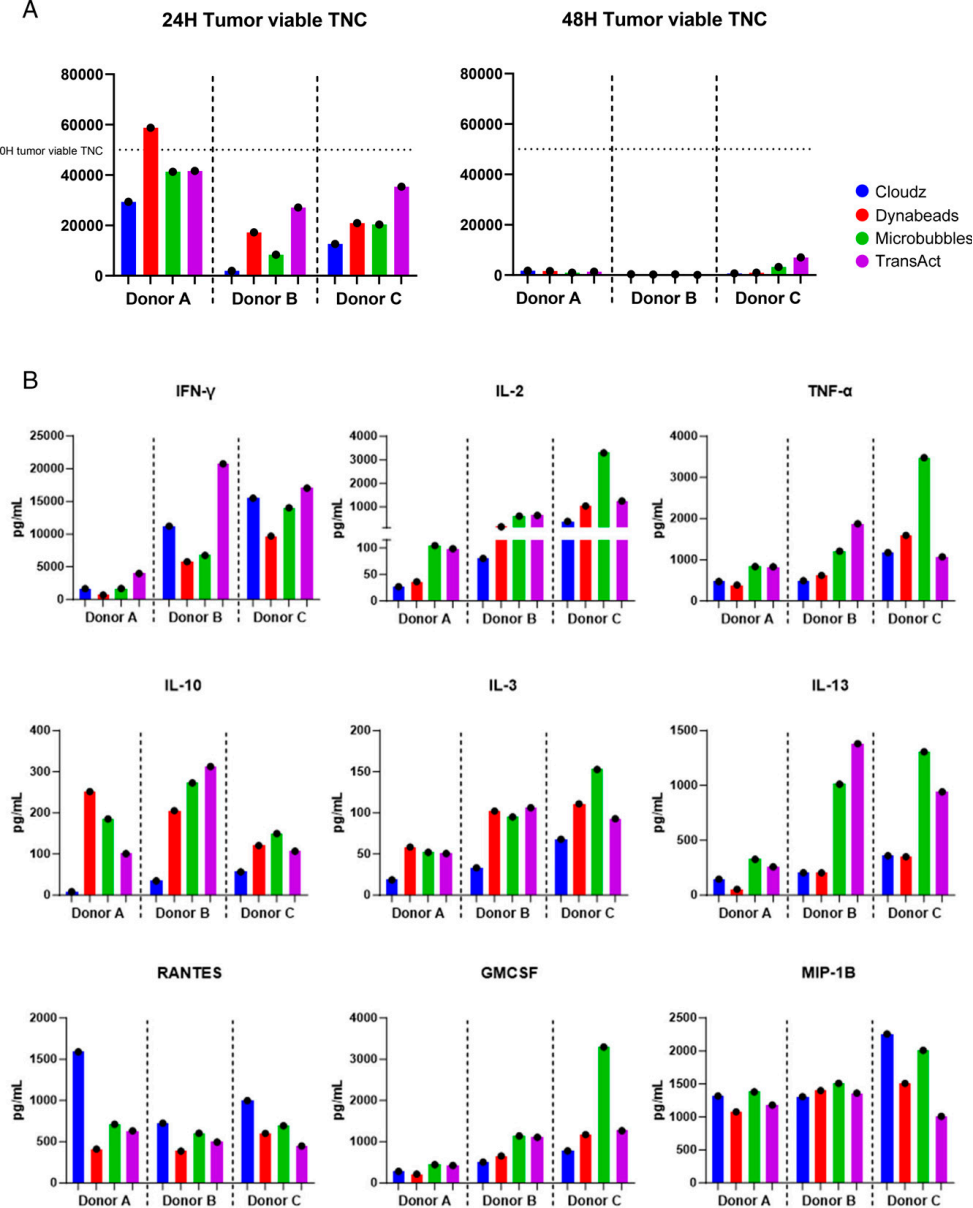
T cell functional assays. (**A**) The tumor cell viable total nucleated cells (TNCs) are shown at the 24-h time point at an E:T cell ratio of 1:1 (left). The seeding tumor cell TNCs are represented with the dotted line at 5 × 10^4^. At the 48-h time point at 1:1 E:T, very few tumor cells remained (right). (**B**) Cytokine protein concentrations were analyzed using the Bio-Plex 200 for 1:1 E:T ratio killing assays.

**FIGURE 5.**
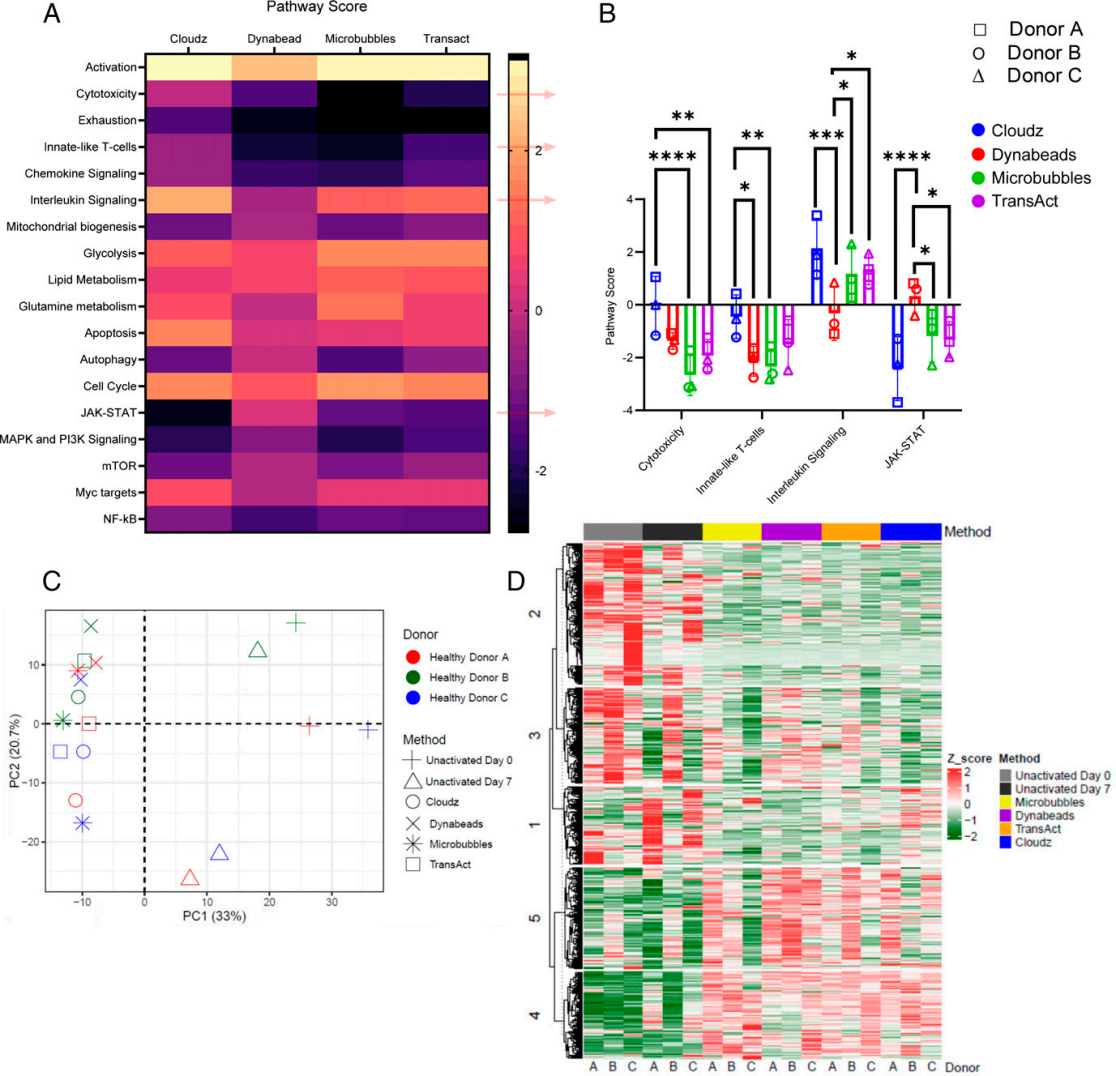
Gene expression profile using the CAR T characterization panel from NanoString nCounter. (**A**) Pathways are listed on the *y*-axis, and activator groups are listed on the *x*-axis. Yellow indicates high scores; black indicates low scores. Scores are displayed on the same scale via a Z-transformation. (**B**) Pathways from (A) with significant differences on the pathway scores among four activator groups. *x*-Axis represents pathways; *y*-axis indicates pathway score. *p ≤ 0.05, **p ≤ 0.01, ***p ≤ 0.001, ****p ≤ 0.0001. (**C**) Principal-component analysis loading plot indicates the distribution of the analyzed genes for each donor/method pair. Percentages represent variance captured by each principal component, 1 and 2. Controls are shown as ▲ (nonstimulated) and + (day 0), and donors are colored differently. (**D**) Gene expression data of 780 genes are shown as a heatmap for each sample. Vertical axis represents differentially expressed genes (DEGs) with log2 gene expression intensity values. Red and green represent upregulated and downregulated genes, respectively. Heatmap is clustered using both gene categories and samples (methods), and gene expression data were scaled by each sample.

GraphPad Prism 9 software was used for statistical analysis. Significance was determined by ANOVA followed by Tukey’s multiple comparisons test for Figs. 1B and 5. Paired *t* tests were used to determine statistical significance for Figs. 1D and 2–4. A *p* value of <0.05 was considered statistically significant. Bars and/or lines on graphs indicate mean with SD unless otherwise indicated or not shown.

## Results

### T cells stimulated by different activators show similar fold expansion, viability, and transduction efficiency

For all four activators, T cells underwent significant expansion in both the transduced groups and the untransduced controls compared with the unactivated control. Fold expansion ranged between 1.08-and 3.14-fold between days 2 and 4, and 4.6- and 8.5-fold between days 4 and 7 (Fig. 1B). Cloudz-stimulated cells had a slightly lower average fold expansion for days 4–7 of 5.5-fold for untransduced cells and 5.7-fold for transduced cells versus 6.5- to 6.9-fold for untransduced cells or 6.8- to 7.6-fold for transduced cells for the three other groups. Significant differences in fold expansion of transduced cells were observed when comparing Cloudz- and Dynabeads-activated groups on days 4–7 (*p* = 0.04). Fold expansion was comparable for untransduced and transduced groups within each activator group. On day 7 of culture, activated cells, both transduced and untransduced, had viabilities averaging between 93.47 and 96.03%, whereas unactivated cells averaged slightly lower at 84.70% cell viability as expected for cells in culture for 7 d without stimulation (Fig. 1C).

Within each activated cell group, there was a high level of donor variability in the transduction efficiency, which was quantified using an anti-CD19 idiotype and expressed as a percentage of all viable CD3 T cells. Because T cells from donors A and B were transduced at MOI 40 and the transduction efficiency was only 10.9–30.6%, we increased the MOI to 80 for donor C, which resulted in an increase in transduction efficiency to 22–56.1%. For each donor, the Cloudz and TransAct groups had lower transduction efficiencies (average of 23.33 and 20.33%, respectively), whereas Dynabeads and Microbubbles activation resulted in higher transduction efficiencies (average of 37.93 and 34.70%, respectively) (Fig. 1D). Paired *t* test analysis showed a significant difference in T cell transduction between Cloudz and Dynabead activators (*p* < 0.05). Interestingly, the higher MOI 80 yielded increases in transduction efficiency for all groups except the TransAct-activated group for donor C.

### Activation and exhaustion marker dynamics between stimulator groups

We measured the surface expression of CD25, PD1, and Lag3 on days 0, 2, 4, and 7 of culture on cells from all donors using flow cytometry. The expression of CD25 by CD3 cells was nearly identical among the four groups at all time points tested, starting at around 20% CD25 expression on day 0 and increasing to >95% CD25 on days 4–7 (Fig. 2A). There were no significant differences between the groups at the different time points (Fig. 2B), and as expected, unactivated controls showed no increase in CD25 expression throughout the culture.

When examining PD1 expression of CD3 cells, we observed more variability between donors and activator groups (Fig. 2A). Generally, baseline PD1 expression was higher at day 0 (between 28.96 and 42.44%), increased substantially on days 2 and 4, and then decreased on day 7. The expression of PD1 was highest among Dynabeads-activated groups (average of 84.15%) on day 2, whereas Cloudz-activated groups expressed PD1 at the lowest level (average of 63.73%), and Microbubbles and TransAct were in between with averages of 74.74 and 75.63%, respectively. A paired *t* test showed a significant difference (*p* < 0.05) between Cloudz and TransAct groups on day 2. The expression of PD1 declined on day 7 in all groups and donors, indicating the cells were returning to their resting state (day 7 averages range from 40.90 to 57.95%). Expression of PD1 by CD3 T cells in unactivated controls remained low throughout the culture period.

Analysis of LAG3 expression by CD3 cells revealed <5% expression at day 0, which increased dramatically on days 2 and 4 before returning to <18% on day 7 (Fig. 2A). On days 2 and 4, Dynabeads stimulation resulted in higher expression of LAG3. On day 2, Dynabeads-stimulated groups expressed LAG3 at the highest level (average of 81.10%), followed by Microbubbles stimulation (72.73%), whereas Cloudz- and TransAct-stimulated groups expressed LAG3 at lower levels comparatively (58.18 and 62.17%, respectively). Significant differences are shown in Fig. 2B. The lack of differences observed in PD1 and LAG3 on day 7 would suggest that exhaustion is likely not a factor in these cultures.

### Effects of activators on T cell subsets

The three healthy donors whose cells were used in this study had a wide range of CD4/CD8 T cell ratios in the starting material used to initiate the CAR T cell culture (0.6, 2.4, and 1.4 on day 0 for donors A, B, and C, respectively) (Fig. 3A). We observed that by day 7 all activators except for Cloudz generated a final CAR T cell product that was skewed toward CD4 T cells, whereas Cloudz-activated groups resulted in an increase in CD8 cells for all donors. For Cloudz, the mean CD4/CD8 ratio was 0.4 on day 7 compared with a range of 2.0–2.3 mean ratio for the other activators (Fig. 3A). Cloudz stimulation resulted in a mean of 26.2% CD4 and 67.9% CD8 CAR T cells on day 7, whereas for CAR T cell products produced with the other activators, the mean percentages ranged from 63.0 to 65.3% CD4 and 31.3 to 33.4% CD8 cells (Fig. 3B). Overall, this meant that Cloudz had a >2-fold increase in percent CD8 cells (average of 2.16-fold) compared with the other conditions within each donor.

When examining T cell differentiation subsets, we found that in each donor, Dynabead stimulation resulted in a significantly higher frequency of T cells with a naive phenotype (CD62L^+^CCR7^+^CD45RA^+^) than Cloudz, Microbubbles, and TransAct. The average fold increase in Tnaive when stimulated with Dynabeads over Cloudz, Microbubbles, and TransAct was 1.98, 1.27, and 2.01, respectively. Microbubble stimulation resulted in a trend toward a higher frequency of central memory T cells in each donor, and Cloudz resulted in a higher frequency of effector memory/effector T cells than all other stimulators, although no significant differences were observed (Fig. 3C).

### Functional attributes of CAR T cells

CAR T cell functional assays were performed by coculturing CAR T cells with NALM6 acute lymphocytic leukemia tumor cell line that expressed CD19 and CD22 on the cell surface (12). Cells were cocultured for 24–48 h, and tumor cell number was enumerated by counting GFP^+^ cells, expressed by NALM6, to evaluate CAR T cell cytotoxicity. At 24 h, we observed the highest percentage of tumor cells killed for Cloudz-stimulated cells from each donor (range 41.2–96.0%). Donor A CAR T cells appeared to have a more limited capacity to kill target cells at 24 h (range 0–41.2% killed) (Fig. 4A). At 48 h, CAR T cell killing of NALM6 tumor cells was observed in CAR T cells produced with all four activators and among all donors. Target cell death was not observed in untransduced control or tumor cell–only groups (data not shown).

Supernatants from the 1:1 E:T cell cocultures were taken at 24 h and analyzed for cytokine secretion. The concentration of IFN-γ was greatest in supernatants from cocultures with TransAct-stimulated CAR T cells from all donors, followed closely by coculture with CAR T cells stimulated with Cloudz (Fig. 4B). Dynabead-stimulated CAR T cells had the lowest IFN-γ levels within all the donors. IL-2 concentrations were highest in cocultures with CAR T cells stimulated with TransAct and Microbubbles (Fig. 4B). TNF-α levels were more variable among donors, having the highest concentration from CAR T cells from donors A and B produced with TransAct and Microbubbles, and the lowest concentration of TNF-α from CAR T cells from donor C with TransAct (Fig. 4B). All other cytokines for which less pronounced trends were observed are shown in Supplemental Fig. 3.

### CAR T cells stimulated by specific activators show differences in gene expression

To explore the transcriptomic differences among the CAR T cells produced with the four different activators, we analyzed gene expression of CAR T cells on day 7 using the NanoString nCounter gene profiling system. Comparison of gene expression pathway scores (Fig. 5A) revealed several marked differences among CAR T cells from the four activator groups. Notably, significant disparities were observed in key pathways: cytotoxicity, innate-like T cells, IL signaling, and JAK-STAT among these activator groups, as depicted in Fig. 5B. Principal-component analysis shows the distribution of the analyzed genes. Similar gene expression patterns are shared among activation groups (Fig. 5C). Fig. 5D illustrates a heatmap of the entire set of 780 differentially expressed genes separated by method. Supplemental Table II shows the clustering of all 780 genes, thus collectively highlighting the distinct pathways that are used upon stimulation by different activators.

## Discussion

Activation and expansion of T cells have been explored using a variety of reagents that expose T cells to anti-CD3 and anti-CD28 (13–17). In this study, we examined the effects on CAR T cell phenotype and function after stimulating these cells at the beginning of the culture with commonly used reagents. We chose to investigate four activator reagents, including Dynabeads (Thermo Fisher), TransAct (Miltenyi Biotec), Cloudz (R&D Systems), and Microbubbles (Diagnologix). Selection of these reagents was based on the fact that their mode of providing T cell stimulation via anti-CD3/anti-CD28 was different, which included magnetic microspheres, polymeric nanomatrix, alginate hydrogel, and lipid membrane containing perfluorocarbon gas, respectively. The CAR used in this study was a CD19/CD22 bivalent CAR lentiviral construct containing a MSCV promoter, CD8 transmembrane sequence, 4-1BB costimulatory domain, and CD3z primary signaling domain. This CAR construct was chosen for these studies because it is currently being used in a series of phase I/II studies at both the National Cancer Institute, NIH and Stanford University for the treatment of children and young adults with recurrent refractory B cell malignancies (https://www.clinicaltrials.gov: NCT03448393, NCT03241940, and NCT03233854) (10, 18). It is important to note that this trial uses the Miltenyi Prodigy instrument and TransAct in the manufacturing process, whereas the studies described in this article are using cell culture bags.

We found many differences between the CAR T cells produced with four different activators. Fold expansion of CAR T cells at harvest (day 7) was significantly less for Cloudz than for Dynabeads, and for Cloudz trended lower than for Microbubbles and TransAct (Fig. 1B).

The expression of activation markers at day 2 poststimulation, represented by expression of CD25^+^ and PD-1^+^ by CD3^+^ cells, was at the highest level among the donors stimulated with Dynabeads. LAG3, which was significantly different between groups on days 2 and 4, again was expressed at the highest level in Dynabead-stimulated cells but returned to the near-baseline levels by day 7 (Fig. 2A, 2B). It is interesting to note that all donors had slower activation kinetics based on all three markers (CD25, PD-1, and LAG3) examined on day 2 using Cloudz when compared with the same donor in the other activation groups. The fact that PD-1 and LAG3 expression decreased in all groups by the end of the culture signified that T cell exhaustion did not appear to play a major role in this short culture period.

Transduction efficiency was consistently higher among all donors for CAR T cells produced with Dynabeads and Microbubbles (Fig. 1D). Transduction efficiency for TransAct-activated groups remained low even after increasing MOI. Another study showed lower transduction efficiency using TransAct in different media and cytokines, compared with Dynabeads (19). The reason for this difference is not clear and merits further investigation.

Upon examining T cell CD4:CD8 ratios, Cloudz reagent had a profound effect on skewing this ratio toward a predominately CD8 population, whereas the other three activators resulted in more CD4 T cells, and the ratios were similar among these three activators. This phenomenon is also generally observed when stimulating PBMCs with anti-CD3 (OKT3) (20). We also noted differences in T cell subtype among CAR T cells produced with the four activators, but the effects of the different activators on T cell subsets were more difficult to decipher given the fact that we observed high donor-to-donor variability in our experiments. All donors had a higher frequency of naive cells at the end of the culture after stimulation with Dynabeads, which was not expected because of the higher-fold CAR T cell expansion and activation that was observed with this activator. There was also a clear increase among all donors in the frequency of effector/effector memory T cells in CAR T cells stimulated with Cloudz; however, this did not reach statistical significance because of the donor variability.

The tumor-killing activity in cocultures was highest in CAR T cells produced with Cloudz for all donors at 24 h, but all CAR T cells killed most tumor cells within 48 h (Fig. 4A). The increased cytotoxicity by Cloudz-stimulated CAR T cells is likely a reflection of the fact that these CAR T cells contained more CD8 T cells, which also correlated with higher levels of IFN-γ secretion and less IL-2 secretion (Fig. 4B). It is interesting that TransAct-stimulated cells also had high levels of IFN-γ in the supernatant, but the cytotoxicity of these cells was much lower, having the lowest killing capacity of the four groups in two of three donors tested. This is in line with recent reports where others have decoupled IFN-γ production from CAR T cell cytotoxicity, and blocking IFN-γ did not result in a decrease in specific lysis of NALM6 cells or in vivo activity (21). IL-10, generally to be considered anti-inflammatory, and IL-3, were reduced in Cloudz-activated cells, whereas RANTES, a proinflammatory cytokine, was elevated. Gene expression profiling revealed several significant differences in pathway scores of the final products. Cloudz-activated cells showed higher overall scores in genes associated with cytotoxicity, innate-like T cells, and IL signaling, but lower scores for genes associated with the JAK-STAT pathway, highlighting clear differences in downstream gene expression between these activators (Fig. 5B).

Between the completion of the experiments and the submission of this manuscript, the R&D Systems Cloudz reagent has been discontinued, but there are other good manufacturing practices–grade activators (Akadeum Life Sciences; STEMCELL Technologies) emerging in the market, as well as some promising good manufacturing practices to be available in production in 2024, including one that claims to allow the user to control the CD4:CD8 ratio in a dose-dependent manner (Nanotein Technologies).

In terms of ease of use of each reagent, because of their relatively large size, the Dynabeads must be magnetically removed from the products prior to administration to the patient, which adds extra time to the process and results in cell loss. The Cloudz reagent must be removed using a specialized disassociation buffer, a step that takes ∼20 min. TransAct is composed of nanoparticles that are washed out during the culture, and there is no debeading step required with this product because they are capable of passing through the kidney; therefore, time and cell recovery are not an issue as in Dynabeads debeading. There is also no debeading step for using the Microbubble activator, because Microbubbles will spontaneously burst within 24 h in cell culture, and the ingredients of Microbubbles (phospholipids and perfluorocarbon) are biologically compatible.

These studies are similar to others that have shown that changes in manufacturing can affect CAR T cell characteristics. In other studies, we have found that CAR T cells manufactured with the same vector, media, activating agent, and cytokines but in different types of culture system also have different characteristics (22). Although we are not certain whether the differences in CAR T cells attributable to the use of the different activators used in this study would affect their in vivo potency or toxicity, other studies have found that changes in manufacturing can affect their in vivo performance. We have found changing the method of enriching the PBMCs for T cells that were used to manufacture clinical CD22 CAR T cells resulted in changes in potency and toxicity (23). The use of anti-CD4 and anti-CD8 to select T cells from PBMCs resulted in more potent and toxic CD22-CAR T cells than less robust T cell enrichment.

These studies demonstrate the importance of developing potency markers for CAR T cell therapies. Changes in manufacturing are often required because of changes in availability of reagents used in manufacturing or when scaling out the production of clinical CAR T cells. Without reliable potency markers, it is not possible to determine whether changes in manufacturing will affect the clinical outcomes of patients receiving CAR T cells.

In conclusion, we tested several T cell activators and gained further insight into the similarities and differences between the CAR T cell phenotype and in vitro function at the time of harvest. Although all activators were capable of generating a significant number of CAR T cells, our studies demonstrate substantial differences in key parameters, such as CD4/CD8 ratio, killing kinetics, and cytokine secretion, that merit consideration when selecting one of these reagents to be used for clinical manufacturing.

## Supplementary Material

Supplemental Material (PDF)

Supplementary Table 1 (CSV)
