# Supplementary material for: T Cell Activators Exhibit Distinct Downstream Effects on Chimeric Antigen Receptor T Cell Phenotype and Function
Source: Immunohorizons. 2024 Jun 10;8(6):404–14. doi: 10.4049/immunohorizons.2400008 (PMC11220740; doi:10.4049/immunohorizons.2400008)
Supplement: Supplemental Material (PDF) [file IH_2400008_Supplemental_1.pdf]

Supplemental Figure 1

A.

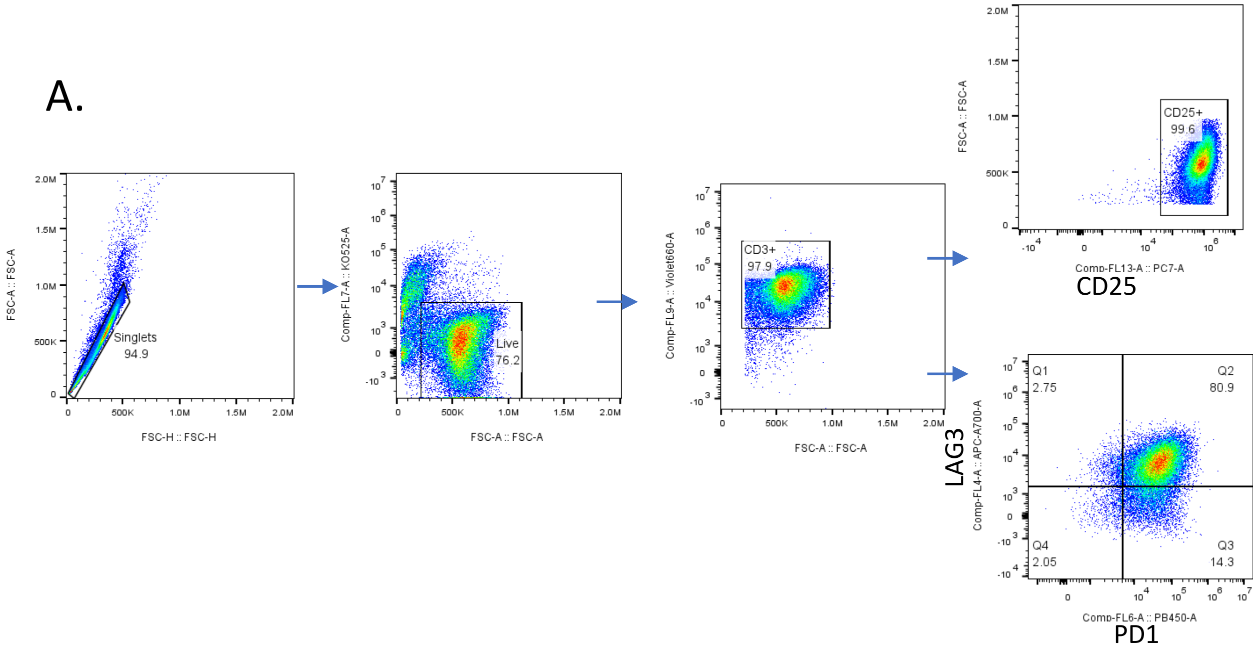

Supplemental Figure 1. Gating Strategy for CD25, LAG3, PD1. A) Cells from Donor C Microbubbles on day 4 were gated on singlets, followed by live cells (7AAD-), CD3+. Surface expression of CD25, LAG3 and PD1 was then gated from CD3+ cells.

Supplemental Figure 2

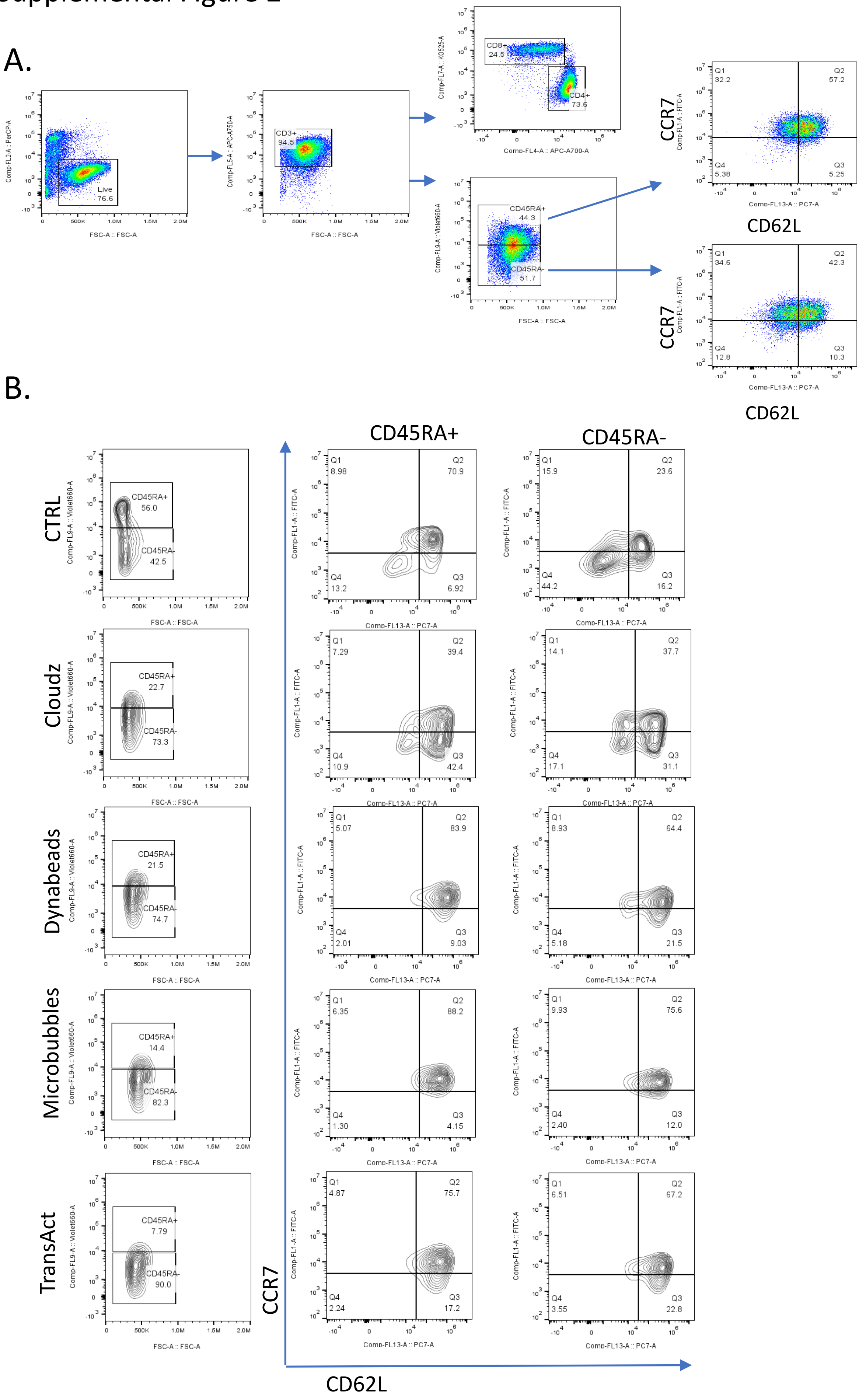

Supplemental Figure 2. Gating strategy for T-cell subsets. A) Cells from Donor C Microbubbles group on day 4 were gating on live cells first, followed by CD3+ cells. CD45RA positive and negative cells were then gated on their expression of CCR7 and CD62L, separately. B) Representative example of this gating strategy as applied to Donor C day 7 groups.

Supplemental Figure 3

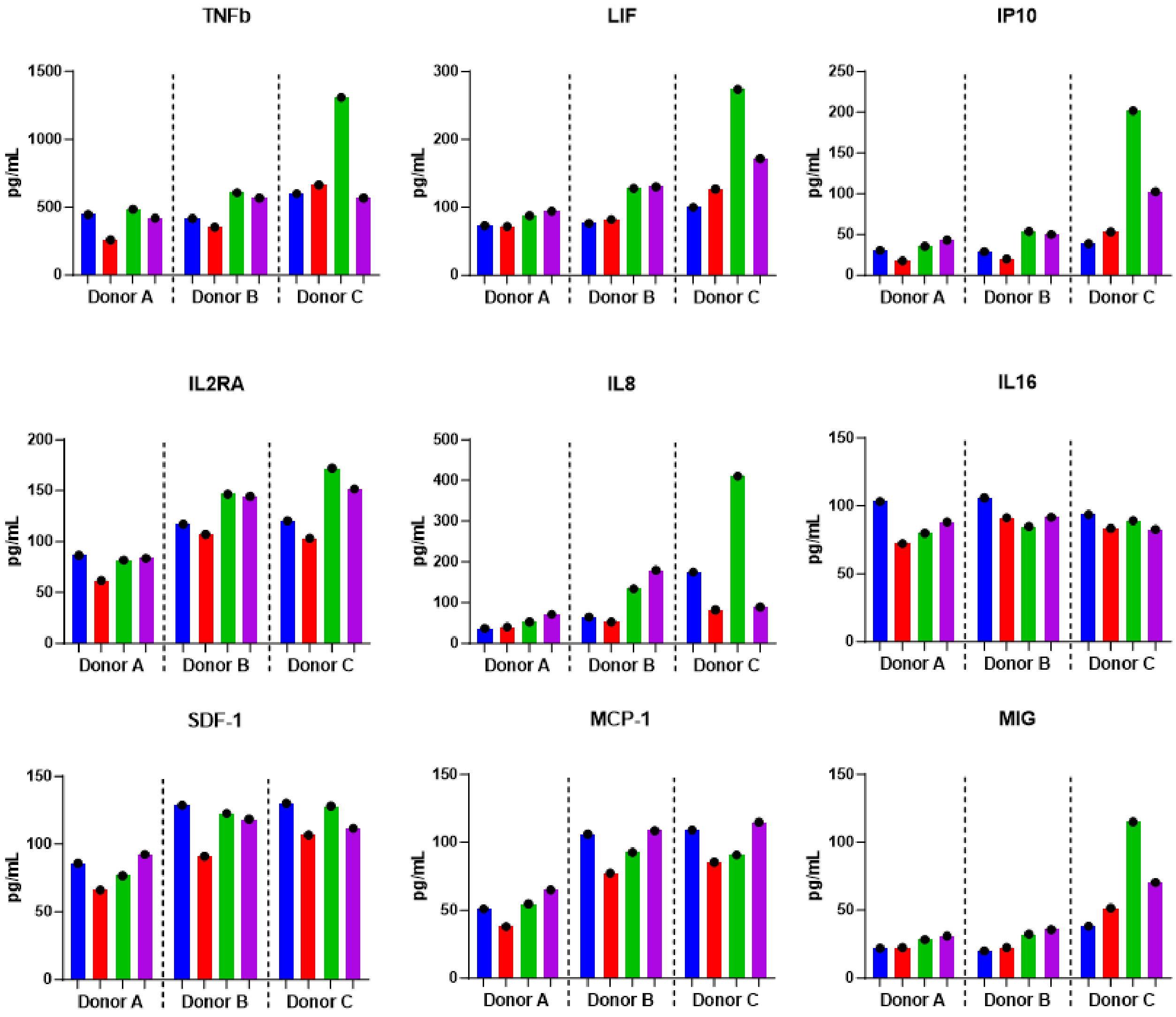

Supplemental Figure 3. Additional Cytokine protein concentrations from the Bio-Plex 200 from 1:1 Effector:Tumor ratio killing assays at 24 hours.

Supplementary Table 1. FACS Antibodies used and their source

Antibody	Supplier	Catalog
FITC Mouse Anti-Human CD8 Clone SK1	BD Biosciences	347313
APC-R700 Mouse Anti-Human LAG-3 (CD223) Clone T47-530	BD Biosciences	565774
BV421 Mouse Anti-Human CD279 (PD-1) Clone EH12.1	BD Biosciences	562516
BV605 Mouse Anti-Human CD4 Clone RPA-T4	BD Biosciences	562658
BV650 Mouse Anti-Human CD3 Clone SK7	BD Biosciences	563999
PE-Labeled Monoclonal Anti-FMC63 scFv Antibody, Mouse IgG1 (Y45)	AcroBio	FM3-HPY53
PE-Cy™7 Mouse Anti-Human CD25 Clone M-A251	BD Biosciences	557741
FITC Mouse anti-Human CD197 (CCR7) Clone 150503	BD Biosciences	561271
Alexa Fluor® 700 Mouse Anti-Human CD4 Clone RPA-T4	BD Biosciences	557922
APC-H7 Mouse Anti-Human CD3 Clone SK7	BD Biosciences	560176
BV510 Mouse Anti-Human CD8 Clone SK1	BD Biosciences	563919
BV650 Mouse Anti-Human CD45RA Clone HI100	BD Biosciences	563963
PE-Cy™7 Mouse Anti-Human CD62L Clone DREG-56	BD Biosciences	565535

Supplemental Table 1. FACS antibodies used and their sources.
